# A Chimeric UDP-Glucose Pyrophosphorylase Produced by Protein Engineering Exhibits Sensitivity to Allosteric Regulators

**DOI:** 10.3390/ijms14059703

**Published:** 2013-05-06

**Authors:** Matías D. Asención Diez, Ana C. Ebrecht, Lucila I. Martínez, Mabel C. Aleanzi, Sergio A. Guerrero, Miguel A. Ballícora, Alberto A. Iglesias

**Affiliations:** 1 Instituto de Agrobiotecnología del Litoral (UNL-CONICET), Facultad de Bioquímica y Ciencias Biológicas, Paraje “El Pozo” CC 242, S3000ZAA Santa Fe, Argentina; E-Mails: masencion@fbcb.unl.edu.ar (M.D.A.D.); anaebrecht@gmail.com (A.C.E.); lucilama@fbcb.unl.edu.ar (L.I.M.); maleanzi@fbcb.unl.edu.ar (M.C.A.); sguerrer@fbcb.unl.edu.ar (S.A.G.); 2 Department of Chemistry and Biochemistry, Loyola University Chicago, 1068 W Sheridan Rd., Chicago, IL 60660, USA; E-Mail: mballic@luc.edu

**Keywords:** protein engineering, allosteric regulation, pyrophosphorylases evolution, UDP-glucose, ADP-glucose

## Abstract

In bacteria, glycogen or oligosaccharide accumulation involves glucose-1-phosphate partitioning into either ADP-glucose (ADP-Glc) or UDP-Glc. Their respective synthesis is catalyzed by allosterically regulated ADP-Glc pyrophosphorylase (EC 2.7.7.27, ADP-Glc PPase) or unregulated UDP-Glc PPase (EC 2.7.7.9). In this work, we characterized the UDP-Glc PPase from *Streptococcus mutans*. In addition, we constructed a chimeric protein by cutting the C-terminal domain of the ADP-Glc PPase from *Escherichia coli* and pasting it to the entire *S. mutans* UDP-Glc PPase. Both proteins were fully active as UDP-Glc PPases and their kinetic parameters were measured. The chimeric enzyme had a slightly higher affinity for substrates than the native *S. mutans* UDP-Glc PPase, but the maximal activity was four times lower. Interestingly, the chimeric protein was sensitive to regulation by pyruvate, 3-phosphoglyceric acid and fructose-1,6-bis-phosphate, which are known to be effectors of ADP-Glc PPases from different sources. The three compounds activated the chimeric enzyme up to three-fold, and increased the affinity for substrates. This chimeric protein is the first reported UDP-Glc PPase with allosteric regulatory properties. In addition, this is a pioneer work dealing with a chimeric enzyme constructed as a hybrid of two pyrophosphorylases with different specificity toward nucleoside-diphospho-glucose and our results turn to be relevant for a deeper understanding of the evolution of allosterism in this family of enzymes.

## 1. Introduction

The fate of Glc-1P in sugar anabolism involves a first step where the Glc moiety is “activated” by the formation of a nucleoside-diphospho-glucose (NDP-Glc) catalyzed by different NDP-Glc pyrophosphorylases (NDP-Glc PPases). Later, diverse glycosyl transferases with specificity toward a particular NDP-Glc lead the monosaccharide to a variety of carbohydrate metabolic routes. In general, in bacteria, there are two major biochemical roles for nucleotide-linked sugars: as intermediates in the formation of monosaccharides used in the production of complex carbohydrates, via UDP-Glc or as glycosyl donors for glycogen synthesis, using ADP-Glc [1,2]. These two key metabolites are products of either UDP-Glc or ADP-Glc PPase, through a reaction that requires a divalent metal ion (physiologically Mg^2+^): U(A)TP + Glc-1P ↔ U(A)DP-Glc + PP_i_. Other specific NDP-sugar PPases complement the metabolic scenario for the production of the multiple mono-, oligo-, and poly-saccharides in the cell, which are found as free components or covalently bound to proteins and lipids [3,4].

UDP-Glc PPase (EC 2.7.7.9) is ubiquitously distributed in all types of organisms, and it plays a critical role in carbohydrates metabolism [5]. Significant differences at the level of amino acids sequence and three-dimensional structure found between the enzymes from prokaryotes and eukaryotes imply that they are not homologous. Eukaryotic UDP-Glc PPases are bigger than those found in bacteria [5,6], and the enzyme from *Entamoeba histolytica* (and probably from all protozoa) was recently characterized as being regulated by redox modification of critical cysteinyl residues [7]. Many bacterial UDP-Glc PPases have been characterized [8–13], and the crystallographic structures of the enzyme from *Escherichia coli* [14], *Sphingomonas elodea* [15] and *Corynebacterium glutamicum* [16] have been elucidated. The prokaryotic UDP-Glc PPase is a dimeric/tetrameric protein formed by a single subunit of ~35 kDa with a relatively high specific activity and specificity for Glc-1P and UTP [8–13].

A main characteristic among prokaryotic NDP-sugar PPases (including UDP-Glc PPase) is that they are non-regulated enzymes. However, ADP-Glc PPase (EC 2.7.7.27) is an exception in that it catalyzes the key regulatory step in the pathway for glycogen and starch biosynthesis in bacteria and plants, respectively. Most ADP-Glc PPases characterized so far are allosterically regulated by metabolites that are principal intermediates in the major carbon assimilation pathway in the respective organism [1,2]. The three-dimensional structure of the homotetrameric forms of the enzyme from potato tuber and *Agrobacterium tumefaciens* has been recently solved by X-ray crystallography [17,18]. Structural studies have determined that ADP-Glc PPases are larger than other prokaryotic NDP-sugar PPases. The former enzymes have an N-terminal catalytic domain (structurally similar to all PPases) that contains the active site, plus an additional C-terminal domain (absent in other PPases). On the basis of different studies [19–25], it has been proposed that the distinctive C-domain in ADP-Glc PPases is functionally related to allosteric regulation. Herein, we report the molecular cloning and heterologous expression of the gene coding for UDP-Glc PPase from *Streptococcus mutans*. Also, we constructed a chimeric protein by fusing to the latter *S. mutans* enzyme the C-terminal domain of the *E. coli* ADP-Glc PPase. The resulting hybrid protein retained UDP-Glc PPase activity and exhibited allosteric properties, being activated by 3-phosphoglycerate (3-PGA), fructose-1,6-bis-phosphate (Fru-1,6-bisP) and pyruvate (Pyr). Our results have an impact on understanding the structure-to-function relationship between domains in PPases as well as the strategic changes driven by evolution to awaken allosterism in proteins.

## 2. Results and Discussion

### 2.1. Isolation and Analysis of the Gene Coding for UDP-Glc PPase in *S. mutans* and Construction of the Chimeric Protein

The genome elucidated for *S. mutans* UA159 indicates the presence of a single gene coding for a putative UDP-Glc PPase (SMU_322c; Gene ID: 1029376) [26]; known as *galU* according to previous reports [6,9,10,27] or *gtaB* in other sources [8,28]. To determine the functional role of *galU* in *S. mutans* we amplified this single gene from *S. mutans* ATCC 25175 using specific primers properly designed (see details under Experimental Section) based on the database information available for *S. mutans* UA159 [26]. The amplified gene (the sequence of which was deposited in NCBI; GenBank accession number KC626324) codes for a protein 100% identical to the one in the genome of the reference strain. The *S. mutans galU* gene codes for a protein (*Smu*GalU) with a theoretical molecular mass of 33.9 kDa and a 33.8% and 40.4% identity with UDP-Glc PPases from *C. glutamicum* and *Helicobacter pylori*, respectively. The former was used to elucidate the active site geometry in this type of enzymes [16] and the *H. pylori* protein for determining the enzymatic reaction mechanism (which was bi-bi ordered) [29]. Also, the protein coded by *S. mutans galU* shares similar identity to the UDP-Glc PPase from *E. coli* (41.5%); *Sphingomonas elodea* (41.1%) and *Streptomyces coelicolor* (43.8%), which have been structurally and kinetically characterized [8,14,15].

**Figure 1:**
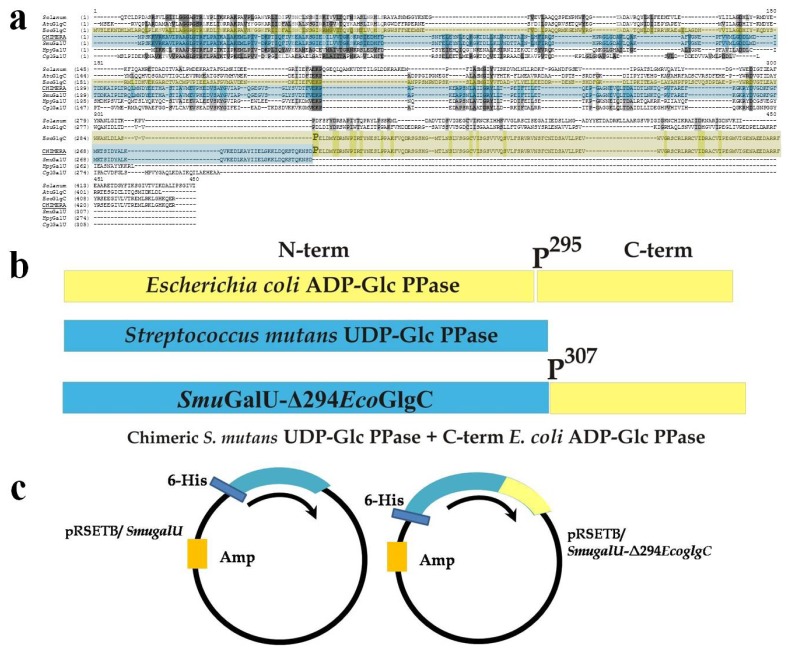
Schematic representation for chimeric *Smu*GalU-Δ294*Eco*GlgC construction. (**a**) Alignment between different ADP-glucose pyrophosphorylases (ADP-Glc PPases) and UDP-Glc PPases (Ref: *Solanum*: ADP-Glc PPase small subunit from potato; *Atu*GlgC, *A. tumefaciens* ADP-Glc PPase; *Eco*GlgC, *E. coli* ADP-Glc PPase; Chimera, chimeric *Smu*GalU-Δ294*Eco*GlgC; *Smu*GalU, *S. mutans* UDP-Glc PPase; *Hpy*GalU, *H. pylory* UDP-Glc PPase; *Cgl*GalU, *C. glutamicum* UDP-Glc PPase). *E. coli* ADP-Glc PPase P^295^ is over-marked; (**b**) Construction of chimeric enzyme: The *C*-terminus belonging to the ADP-Glc PPase from *E. coli* (yellow) was “added” to the entire UDP-Glc PPase from *S. mutans* ATCC 25175 (light-blue); (**c**) Plasmids used to express both proteins.

The *S. mutans* GalU has a high identity (85.1%) with the UDP-Glc PPase from *S. pneumoniae* [9,27]. The gene amplified from *S. mutans* ATCC 25175 was utilized for two main purposes (Figure 1): (i) to insert it into the pRSET-B expression plasmid, looking to produce the recombinant UDP-Glc PPase (*Smu*GalU) as a tool for the structural and kinetic characterization; and (ii) to construct a gene coding for a chimeric protein, seeking to investigate the functionality of key domains in PPases. Amino acid sequence alignment between UDP-Glc and ADP-Glc PPases shows that these proteins share a homologous N-terminal domain, which is involved in catalysis. However, the enzymes specific for ADP-Glc are larger proteins with an extra C-terminal domain that is presumably related to allosteric properties as indicated above (Figure 1a, amplified in Figure S1). To advance in the latter hypothesis, we produced a hybrid protein by fusing the putative regulatory C-domain (from the *E. coli* ADP-Glc PPase) to the C-terminal of the non-allosteric UDP-Glc PPase from *S. mutans* (Figure 1b).

Figure 1 details the “cut and paste” strategy used to construct the chimeric gene coding for the hybrid protein *Smu*GalU-Δ294*Eco*GlgC. Hence, we pasted a DNA fragment cut from the *E. coli glgC* gene, which codes for the 137 C-terminal amino acidic residues of the ADP-Glc PPase (starting at codon belonging to P^295^ residue, which is in a connector loop) to the entire *S. mutans galU* gene. The resulting 1329 pb DNA piece was used to construct a pRSET-B derivative plasmid (Figure 1c) suitable to express a 443 amino acid chimeric protein with a theoretical molecular mass of 49.4 kDa. Previously chimeric enzymes were obtained and characterized after switching/swapping N- and C-terminal domains between ADP-Glc PPases (specifically involving the enzymes from *E. coli* and *A. tumefaciens* [19] or from prokaryotic and eukaryotic photosynthetic organisms [30]). This is the first report regarding a hybrid UDP-Glc PPase/ADP-Glc PPase protein.

### 2.2. Structural and Kinetic Characterization of *Smu*GalU and Chimeric *Smu*GalU-Δ294*Eco*GlgC

**Figure 2:**
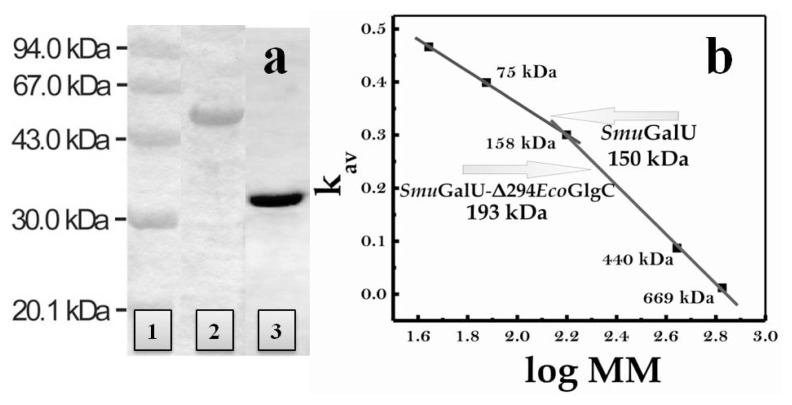
(**a**) Sodium dodecyl sulfate polyacrylamide gel electrophoresis (SDS-PAGE) of recombinant UDP-Glc PPase from *S. mutans* and chimeric *Smu*GalU-Δ294*Eco*GlgC after purification. Lane 1: Molecular weight markers; Lane 2: chimeric *Smu*GalU-Δ294*Eco*GlgC; Lane 3: His-tagged *Smu*GalU. Purifications were conducted as described in the Experimental Section; (**b**) Molecular mass determination, performed from size exclusion chromatography, as detailed in the Experimental Section.

Both *Smu*GalU and chimeric *Smu*GalU-Δ294*Eco*GlgC were produced as recombinant proteins via heterologous expression in *E. coli* using the respective pRSET-B derivative plasmid (see details in Figure 1). Thus, both proteins were obtained having a His-tag fused at the N-terminus. When *E. coli* BL21 (DE3) was used as a host, native *Smu*GalU was over-expressed in soluble fractions but *Smu*GalU-Δ294*Eco*GlgC was mostly recovered in the pellet (inclusion bodies). The chimeric protein appeared in soluble fractions when the expression host was turned to *E. coli* BL21 (DE3) pLysS. Culture conditions and procedures were similar for both hosts (see Experimental Section). After inducing expression and obtaining crude extracts from the transformed cells, the recombinant His-tagged proteins were purified by immobilized metal (Ni^2+^) affinity chromatography, after which they reached a high degree of purity according to sodium dodecyl sulfate polyacrylamide gel electrophoresis (SDS-PAGE) analysis (Figure 2a). Size exclusion chromatography on Superdex 200 revealed that under soluble conditions *Smu*GalU and *Smu*GalU-Δ294*Eco*GlgC arranged homotetrameric quaternary structures of a molecular mass of ~150 kDa and ~200 kDa, respectively (Figure 2b). Results obtained with *Smu*GalU are in good agreement with tetrameric structures previously determined for UDP-Glc PPases from different sources, e.g., both crystallized enzymes from *E. coli* and *S. elodea* [14,15]. On the other hand, the native form found for *Smu*GalU-Δ294*Eco*GlgC indicates that this chimeric protein shares similar oligomeric properties to the polypeptides that form its hybrid structure.

**Figure 3:**
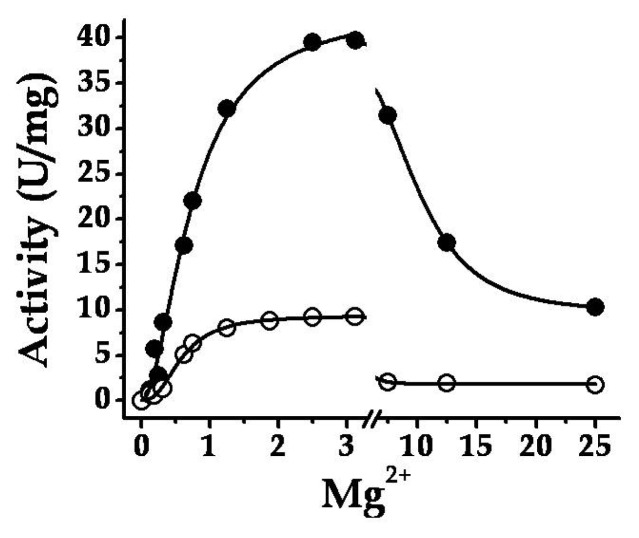
Mg^2+^ curves for both *Smu*GalU (filled circles) and chimeric *Smu*GalU-Δ294*Eco*GlgC (empty circles).

**Figure 4:**
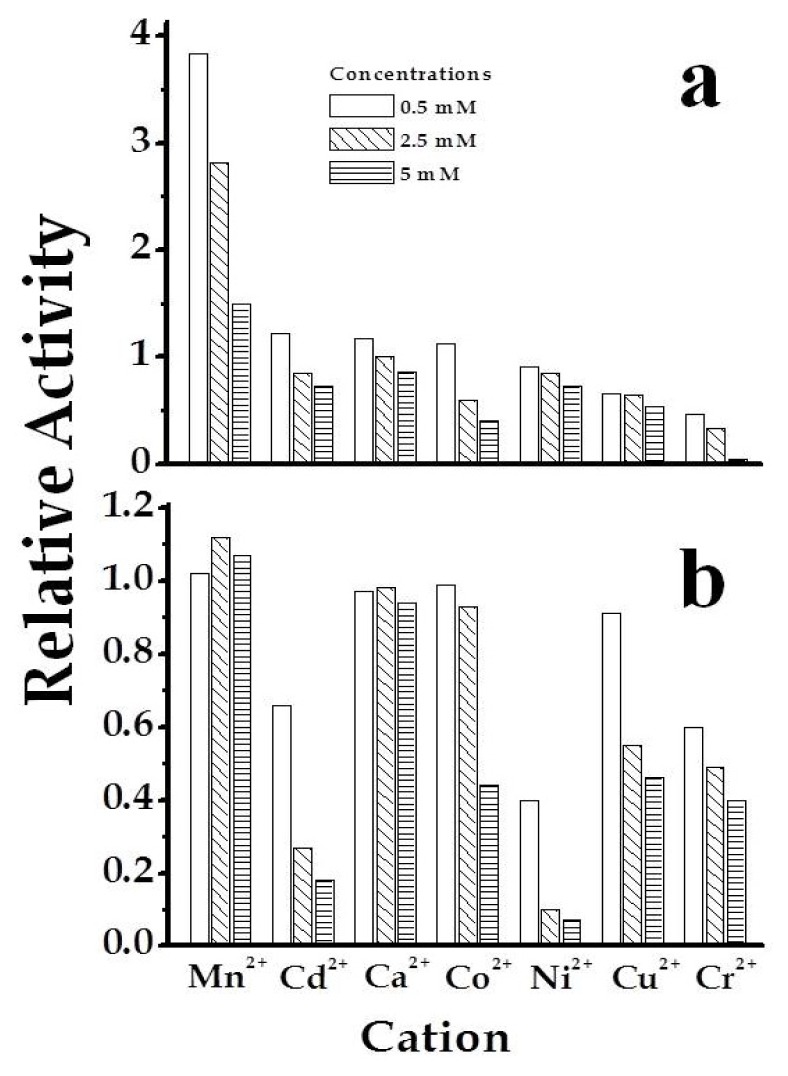
Use of different divalent metal ions by *Smu*GalU (**a**) and chimeric *Smu*GalU-Δ294*Eco*GlgC (**b**). Empty bars correspond to 0.5 mM, sparse filled bars to 2.5 mM and dense filled bars to 5 mM of the corresponding metal analyzed. Controls are related to the enzyme activity measure in the same conditions, using 3 mM Mg^2+^.

Purified *Smu*GalU and *Smu*GalU-Δ294*Eco*GlgC had UDP-Glc PPase activity. It catalyzed the synthesis of UDP-Glc and PP_i_ from Glc-1P and UTP (in the presence of 3 mM Mg^2+^) with specific activities of 40 and 11 U/mg, respectively. It is well known that a divalent metal ion (commonly Mg^2+^) is an essential cofactor for ADP-Glc PPases [1,2] and UDP-Glc PPases [5,8,10,11,16]. In our hands, the activities of *Smu*GalU and *Smu*GalU-Δ294*Eco*GlgC were strictly dependent of Mg^2+^. Both proteins were fully active at ~3 mM and inhibited by higher concentrations of the divalent cation (Figure 3). Other metal ions could replace Mg^2+^, as illustrated by Figure 4. At 0.5 mM Mn^2+^*Smu*GalU reached a four-fold higher activity than with 3 mM Mg^2+^, but the enzyme was inhibited at higher Mn^2+^ levels. This enzyme was also active with Cd^2+^, Ca^2+^, Co^2+^, Ni^2+^, Cu^2+^ and Cr^2+^, with higher activity at 0.5 mM of the metal ion and inhibition at different levels with higher amounts of the respective divalent cofactor (Figure 4a). Concerning *Smu*GalU-Δ294*Eco*GlgC, the protein was as active as with 3 mM Mg^2+^ when assayed with 0.5 mM of Mn^2+^, Ca^2+^, Co^2+^, or Cu^2+^, whereas with 0.5 mM Cd^2+^, Ni^2+^, or Cr^2+^ it showed only about half the activity. Except for Mn^2+^ and Ca^2+^, the other divalent cations inhibited the hybrid protein at higher concentrations (Figure 4b).

A similar pH-dependence of activity was observed for *Smu*GalU and *Smu*GalU-Δ294*Eco*GlgC when studied in the range of pH 6.0–10.0. Both proteins exhibited a maximum at pH 8.0, and sharply decreased below pH 7.0 (data not shown). These results are in good agreement with those previously reported for pneumococcal UDP-Glc PPase, which is fully active at pH 8.0–8.5 [10], as well as for ADP-Glc PPases from different sources, where optimal activity is around pH 8.0 [1,2]. After the behavior of the proteins in respect to pH and the requirement for divalent metal ions were studied, the further kinetic characterization was performed at pH 8.0 and 3 mM Mg^2+^ as its saturating concentration. The kinetic parameters of *Smu*GalU and *Smu*GalU-Δ294*Eco*GlgC for the substrates Glc-1P, UTP and divalent cofactor Mg^2+^ are summarized in Table 1. The analysis of these parameters is needed to compare the kinetic properties of these recombinant proteins and understand the functionality of the different structure domains in PPases.

*Smu*GalU slightly deviated from a hyperbolic behavior for both UTP and Glc-1P displaying positive cooperativity. Saturation curves for Mg^2+^ were even more sigmoidal (Table 1). Apparent affinities for the different substrates/cofactor exhibited by *Smu*GalU were in the same order of magnitude as those reported for other bacterial UDP-Glc PPases so far characterized [8,10,11,31,32]. The recombinant enzyme reached a *V*_max_ of 62 U/mg (Table 1), which was significantly higher than the UDP-Glc PPases from *E. coli* [32], *S. elodea* [31] and *S. pneumoniae* [10], although not as high as UDP-Glc PPase from *S. coelicolor* [8] and in the same order as the enzyme from *Xanthomonas* spp. [11]. Concerning *Smu*GalU-Δ294*Eco*GlgC, **s**aturation curves also showed a slight or marked deviation from the hyperbolic behavior for the substrates or the divalent cation cofactor, respectively (Table 1). The chimeric protein increased three-fold in the apparent affinity for UTP regarding the UDP-Glc PPase from *S. mutans*, whilst affinities for Glc-1P and Mg^2+^ remained at the same level. Besides, the chimeric protein exhibited a four-fold lower *V*_max_ when compared with *Smu*GalU (Table 1). Then, the results suggest that the C-terminal domain from the fusion of the *E. coli* ADP-Glc PPase to the *S. mutans* UDP-Glc PPase modifies the enzyme to acquire a conformation with slightly reduced ratio *V*_max_/*S*_0.5_ (analogous to ratio *V*_max_/*K*_m_, defined as catalytic efficiency for hyperbolic kinetics) (Table 1). The kinetic properties exhibited by *Smu*GalU-Δ294*Eco*GlgC are remarkable for a hybrid protein composed by domains of PPases with different specificity. Although our studies are the first dealing with kinetic characterization of an UDP-Glc PPase/ADP-Glc PPase chimeric enzyme, they can be compared with previous hybrids between ADP-Glc PPases. Thus, when *N*- and *C*-terminal domains from the *E. coli* and *A. tumefaciens* ADP-Glc PPases were switched, the chimeric construct exhibited activities between 20% and 30% of the value of the original wild-type enzymes [19].

In general, prokaryotic NDP-sugar PPases are relatively specific for their substrates, although some exceptions have been reported. For example, dTTP is a substrate in some bacterial UDP-Glc PPases [8,11,32], whereas GDP-mannose (GDP-Man) PPases from *M. tuberculosis* and from *Leptospira interrogans* exhibit promiscuity in the use of the nucleotide triphosphate (NTP) substrate [33,34]. Different NTPs (ATP; UTP; ITP; GTP; CTP; dTTP) at 0.1, 0.5 and 2.5 mM final concentration were assayed as alternative substrates for *Smu*GalU and *Smu*GalU-Δ294*Eco*GlgC. It was observed that *S. mutans* UDP-Glc PPase was active with dTTP as an alternative to UTP. The activity in the synthesis direction of dTDP-Glc was four-fold lower than the production of UDP-Glc, although the enzyme showed similar affinity for either NTP (*S*_0.5_ 0.54 mM, at 1 mM Glc-1P and 3 mM Mg^2+^). Results with *Smu*GalU are in accordance with previous reports indicating that these enzymes (from a prokaryotic source) are capable of utilizing dTTP [8,11,32]. Conversely, chimeric *Smu*GalU-Δ294*Eco*GlgC exhibited a high specificity toward UTP.

### 2.3. The Chimeric *Smu*GalU-Δ294*Eco*GlgC Protein Exhibits Allosteric Properties

**Figure 5:**
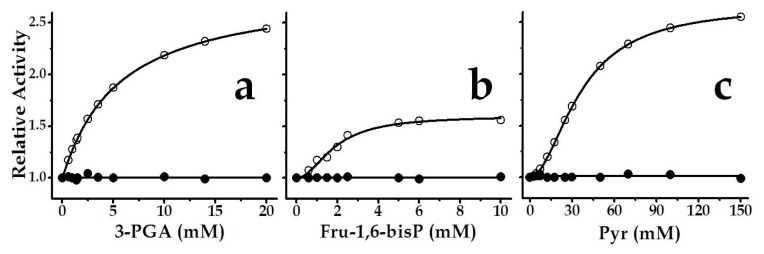
Saturation curves for chimeric *Smu*GalU-Δ294*Eco*GlgC effectors: (**a**) 3-PGA, (**b**) Fru-1,6-bisP and (**c**) Pyr. Filled circles belong to *Smu*GalU and empty circles to chimeric *Smu*GalU-Δ294*Eco*GlgC enzyme. Reactions were conducted at 1 mM Glc-1P, 1 mM UTP and 3 mM Mg^2+^. Values of relative activity were calculated based on activities measured in the absence of effector, specifically 40 U/mg and 11 U/mg for *Smu*GalU and *Smu*GalU-Δ294*Eco*GlgC, respectively.

Activation-inhibition assays were performed for both recombinant proteins under study (*Smu*GalU and *Smu*GalU-Δ294*Eco*GlgC) testing several compounds known to be important effectors of ADP-Glc PPases from different sources [1,2]. The metabolites utilized were phospho*enol*pyruvate (PEP); fructose-1,6-bisphosphate (Fru-1,6-bisP), pyruvate (Pyr), 3-phosphoglyceric acid (3-PGA), Glc-6P, ribose-5P (Rib-5P), fructose-6P (Fru-6P), Man-1P, Man-6P, P_i_, AMP, ADP, NAD^+^, NADH, NADP^+^ and NADPH. These effectors were tested at up to 10 mM (Pyr was even varied up to 150 mM) under activity assay conditions that were saturating and non-saturating in respect to the amount of substrates in the medium. None of the metabolites analyzed affected the activity of *Smu*GalU. This insensitivity to regulation is in good agreement with data reported for bacterial UDP-Glc PPases, which in general are not regulated by allosteric effectors [5]. On the other hand, Pyr, Fru-1,6-bisP and 3-PGA activated chimeric *Smu*GalU-Δ294*Eco*GlgC (Figure 5) (none of the molecules analyzed inhibited the enzyme). This fact *per se* is one of the striking results of this work, since it constitutes the first report regarding a protein having UDP-Glc PPase activity (or a PPase activity other than ADP-Glc PPase) and being subjected to allosteric regulation.

Saturation curves for each metabolite activating *Smu*GalU-Δ294*Eco*GlgC are detailed in Figure 5. Both, 3-PGA and Pyr, increased the *V*_max_ of the hybrid fused protein by 2.4- and 2.6-fold, respectively, while Fru-1,6-bisP activation was 1.6-fold. Respective to the apparent capacity for binding of the allosteric effector, the chimeric enzyme behaved with the highest apparent affinity for Fru-1,6-bisP (*A*_0.5_ 2.0 mM), and with positive cooperativity (*n*_H_ 1.5). For 3-PGA the behavior was hyperbolic (*n*_H_ 1.0) with an *A*_0.5_ value of 5.4 mM, whereas Pyr depicted a sigmoidal (*n*_H_ 1.5) saturation curve from which an *A*_0.5_ of 30 mM could be calculated (Figure 5). It is worth noting that this apparent affinity value of the chimeric protein calculated for Pyr is in the same order as what was found for the interaction (also activating) of this metabolite with the *E. coli* ADP-Glc PPase [19]. 3-PGA and Fru-1,6-bisP are the main activators of plants and some bacterial ADP-Glc PPases [1,35] as well, although with higher affinities.

**Figure 6:**
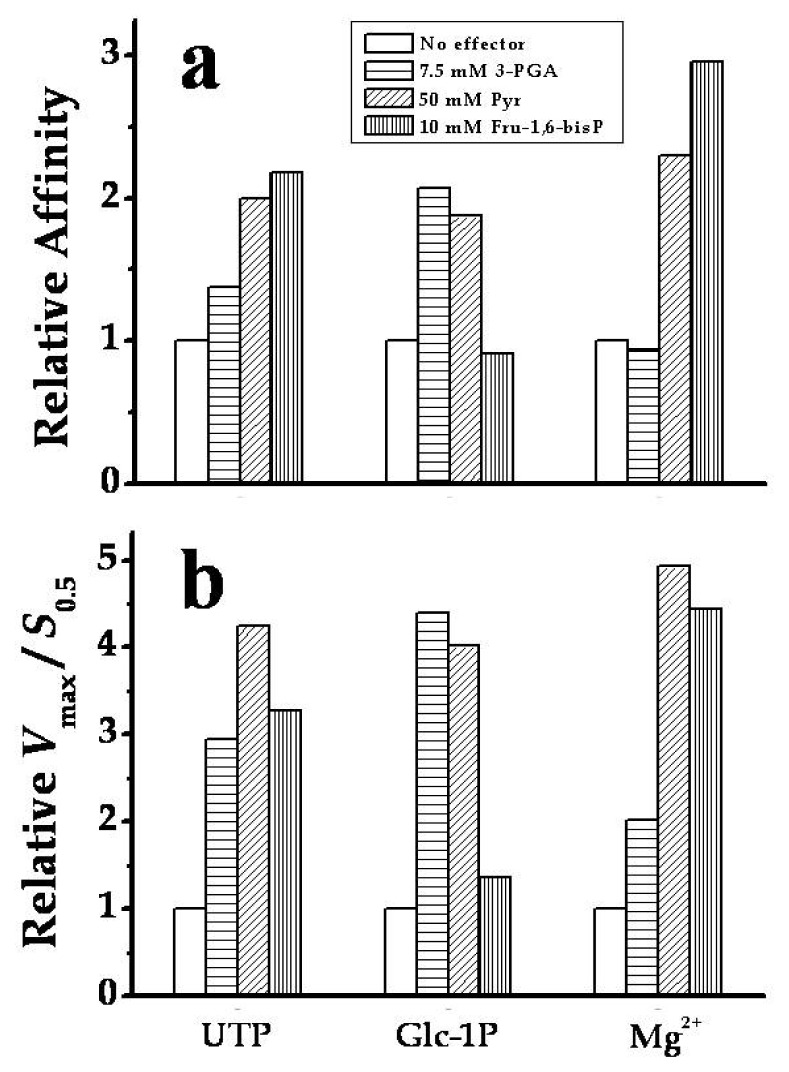
Modification by allosteric activators of *Smu*GalU-Δ294*Eco*GlgC (**a**) substrates apparent affinities; (**b**) catalytic efficiencies (*V*_max_/*S*_0.5_). The enzyme was analyzed with no effector (white bars); 7.5 mM 3-PGA (horizontal line bar), 50 mM Pyr (oblique line bar) or 10 mM Fru-1,6-bisP (vertical line bar). Relative affinity in (**a**) measures the ratio between the *S*_0.5_ values determined in the absence over that in the presence of the stated amount of effector.

It was valuable to analyze the kinetic parameters of *Smu*GalU-Δ294*Eco*GlgC toward the substrates and the divalent ion cofactor when assayed in presence of each allosteric activating compound (Figure 6). Thus, saturation curves for UTP, Glc-1P and Mg^2+^ were conducted in presence of either 7.5 mM 3-PGA, 50 mM Pyr or 10 mM Fru-1,6-bisP. Figure 6a shows how the relative apparent affinity for each substrate/cofactor was affected by the respective allosteric activator. In general, the allosteric effectors decreased the *S*_0.5_ values (increasing the apparent affinity) for substrates of the chimeric enzyme. Notably, Pyr and Fru-1,6-bisP doubled the apparent affinity of the hybrid protein for UTP (the latter with an increment in the sigmoidal behavior). Pyr and 3-PGA also doubled the relative affinity for Glc-1P. On the other hand, the affinity of the chimeric enzyme for Mg^2+^ was not affected by 3-PGA, but it was augmented two- to three-fold by Pyr or Fru-1,6-bis P (Figure 6a). Also, the *V*_max_ of the chimeric enzyme in the absence of an allosteric effector (15.4 U/mg, see Table 1) was enhanced to 24.6, 37.0 or 40.1 U/mg by Fru-1,6-bisP, Pyr or 3-PGA, respectively. The combination of the increase in affinity and of *V*_max_ exerted by the allosteric effectors on the chimeric enzyme determines that they enhance the ratio *V*_max_/*S*_0.5_, which measures catalytic efficiency (Figure 6b), between two- and five-fold. These results highlight the sensitivity to allosteric effectors acquired in the chimeric *Smu*GalU-Δ294*Eco*GlgC.

**Figure 7:**
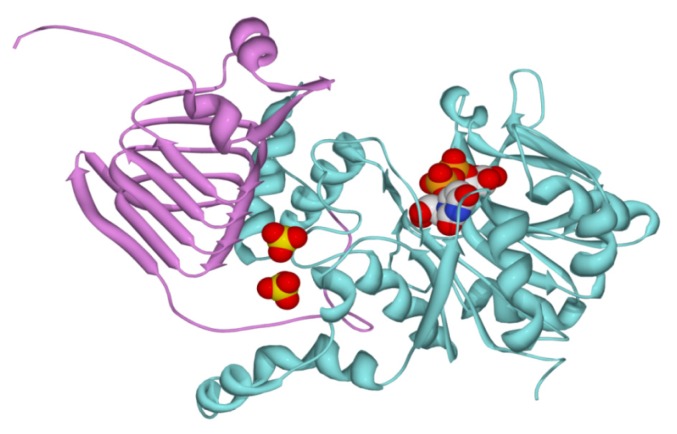
Modeling of the chimeric enzyme. The model was obtained as indicated in the Experimental Section. The UDP-Glc PPase domain from *S. mutans* is depicted in cyan, whereas the C-domain from *E. coli* ADP-Glc PPase is depicted in pink. Two sulfates modeled from the structure of the potato tuber ADP-Glc PPase are shown, together with the product UDP-Glc.

On the basis of the amino acid sequence alignment shown in Figure S2, we constructed a homology model for the chimeric *Smu*GalU-Δ294*Eco*GlgC protein (Figure 7). The structure was modeled using four simultaneous templates. Two templates representing the known atomic coordinates of prokaryotic (*C. glutamicum* and *H. pylori*) UDP-Glc PPases modeled the *Smu*GalU domain from the N-terminus of the chimeric protein. The *C. glutamicum* UDP-Glc PPase structure particularly allowed the location of the product UDP-Glc in the model. Then again, the architecture of the C-terminal domain of the hybrid protein (Δ294*Eco*GlgC) was fitted from the templates corresponding to known structures determined for two ADP-Glc PPases (the enzyme from *A. tumefaciens* and the small subunit from the potato tuber enzyme). The *A. tumefaciens* structure was mostly good for this, since it is a prokaryotic enzyme like *E. coli* GlgC, whereas the potato tuber structure was useful to locate the allosteric ligand in the chimeric protein. Interestingly, the model depicted in Figure 7 shows consistency with the biochemical properties determined for the chimeric protein. Thus, the predicted structure arranges a correct folding for an UDP-Glc PPase catalytic domain (including a UDP-Glc binding site) as well as a functional spatial distribution of the C-term allosteric domain, resembling its allocation in ADP-Glc PPases (Figure 7). When the C-domain is added to the N-terminal domain, it creates a pocket that could serve to accommodate binding to small molecule regulators. This is illustrated by the modeling of two sulfates that in the potato tuber enzyme are located in the inhibitory phosphate site interacting with residues in the C-terminus that are responsible for activation [18].

## 3. Experimental Section

### 3.1. Chemicals

Antibiotics, isopropyl-β-thiogalactoside (IPTG), oligonucleotides, UTP, Glc-1P, 3-PGA, Fru-1,6-bisP and Pyr were obtained from Sigma-Aldrich (St. Louis, MO, USA). All other chemicals were of the highest quality available.

### 3.2. Bacteria and Plasmids

*E. coli* Top 10 F’ cells and pGEM^®^T Easy vector were used for cloning purposes. Expression of *galU* and q*galU* was performed using pRSET-B vector (Invitrogen, Carlsbad, CA, USA) and *E. coli* BL21 (DE3) as host. In addition, *galU* was also expressed using pET24 vector (Novagen, Madison, WI, USA). DNA manipulations and *E. coli* cultures as well as transformations were performed according to standard protocols [36].

### 3.3. Amplification of *galU* Gene from *S. mutans* and Construction of Chimeric *galU*

The 921 pb gene coding for UDP-GlcPPase, *SmugalU*, was amplified using *S. mutans* ATCC 25175 genomic DNA as a template and gene specific Smu1/Smu2 primers, designed according to available information in the GenBank database [37] for *S. mutans* UA159 for gene coding UDP-Glc PPase (Gene ID: 1029376). All oligonucleotides used are detailed in Table 2. The 1350 pb gene coding for the chimeric protein was obtained by overlap extension PCR [38]. In a first step, two independent PCR reactions were conducted, using the pair of primers Smu1/Qmr2 (reaction 1) and the pair Qmr1/Qmr3 (Reaction 2). Both Qmr1 and Qmr2 primers were designed in order to hybridize *SmugalU* and *EcoglgC* region, respectively, starting at the codon for Pro^295^, with extra nucleotides to allow fragment fusion. Reaction 1 was conducted using a plasmid harboring *SmugalU*, while Reaction 2 was used as a template for the plasmid containing the *glgC* gene from *E. coli*. Products from Reaction 1 and Reaction 2 were purified and then were used together as templates in a final PCR step, using Smu1/Qmr3 primers to obtain the complete chimeric gene.

All PCR reaction mixtures (50 μL) contained 100 ng of genomic DNA, 2 pg of each primer; 0.2 mM of each dNTP; 1.5 mM Mg^2+^ and 1U *Taq* or *Pfu* DNA polymerase (Fermentas, St. Leon-Rot, Germany). Standard conditions of PCR were used for 30 cycles: denaturation at 94 °C for 1 min; annealing at 55 °C for 1 min and extension at 72 °C for 2 or 4 min (depending if *Taq* or *Pfu* were used, respectively) with a final extension of 10 min at 72 °C. PCR reaction mixtures were electrophoretically defined in a 1% (*w*/*v*) agarose gel and purified with Wizard SV gel & PCR Clean Up system (Promega, Fitchburg, WI, USA). according to the manufacter’s instructions.

### 3.4. Cloning of *SmugalU* and Chimeric *SmugalU*-Δ294*EcoglgC* Genes

Amplified genes were cloned into the T-tailed plasmid pGEM-TEasy and identities were confirmed by DNA sequencing. The *SmugalU* gene was sub-cloned into pRSET-B *Bam*HI/*Eco*RI sites to achieve a His-tagged protein at N-terminal. The chimeric gene *SmugalU*-Δ294*EcoglgC* was inserted between *Bam*HI/*Xho*I from pRSET-B. Thus, both *Smu*GalU and the *Smu*GalU-Δ294*Eco*GlgC enzyme could be obtained with an N-terminal His-tag (see Figure 1c).

### 3.5. Enzymes Expressions and Purifications

Plasmid harboring *SmugalU* was used to transform *E. coli* BL21 (DE3) competent cells. Transformed cells were grown in YT2X medium at 37 °C, 200 rpm, until the A_600 nm_ reached 0.6–0.8 and were induced with 0.8 mM IPTG at 25 °C overnight. Instead, soluble expression of chimeric *Smu*GalU-Δ294*Eco*GlgC was achieved using *E. coli* BL21 (DE3) pLysS as a host strain and inducing with 0.4 mM IPTG at 25 °C overnight in LB medium.

For expressing purposes, 1 L cultures in conditions detailed above were grown for each protein. Cells were harvested by centrifugation at 5000 rpm for 10 min at 4 °C and resuspended in 5 mL of *buffer H* (20 mM Tris-HCl pH 8.0, 500 mM NaCl, 10 mM imidazole) per g of cells. Supernatants were obtained after cell disruption by sonication on ice, eight times for 30 s with 60 s intervals and centrifugation at 16,000 rpm for 20 min at 4 °C.

*Smu*GalU and chimeric *Smu*GalU-Δ294*Eco*GlgC were expressed as *N*-terminus His-tag fusions, in order to facilitate their subsequent purification. Enzymes were purified by affinity chromatography, using Ni-NTA Agarose resin (Invitrogen, Carlsbad, CA, USA) according to the protocol supplied by the manufacturer. Briefly, crude extract fractions prepared in *buffer H* were loaded onto previously equilibrated columns. After extensively washing with *buffer H*, samples were eluted by means of a lineal gradient to *buffer I* (20 mM Tris-HCl pH 8.0, 500 mM NaCl, 300 mM imidazole). Elution fractions containing the corresponding recombinant enzyme were analyzed by SDS-PAGE [39] to check for purity. For each recombinant enzyme, active fractions eluted from the Ni-NTA column were pooled, dialyzed to remove imidazole and supplemented with 10% (*v*/*v*) glycerol. Both recombinant enzymes were stable for at least six months when stored at −80 °C under the above specified respective conditions.

### 3.6. Protein Methods

Protein concentration was determined by the modified Bradford assay [40] using bovine serum albumin as a standard. Recombinant proteins and purification fractions were defined electrophoreticaly in sodium dodecyl sulphate polyacrylamide gels (SDS-PAGE) according to [39]. Gels were stained with CoomassieBrilliant Blue.

### 3.7. Determination of Activity Optimal pH

Bis-Tris-propane [2,2′-(Propane-1,3-diyldiimino)bis[2-(hydroxymethyl)propane-1,3-diol] (Sigma, St. Louis, MO, USA), which has a wide buffering range (from pH 6.0 to pH 10.0), and tricine (Sigma, St. Louis, MO, USA) *N*-(2-Hydroxy-1,1-bis(hydroxymethyl)ethyl)glycine (from pH 7.5 to pH 9.0) were used to calculate the optimal pH for *Smu*GalU and chimeric *Smu*GalU-Δ294*Eco*GlgC activities. Measures were conducted in the UDP-Glc synthesis way.

### 3.8. Molecular Mass Determination

The molecular mass of the *Smu*GalU and the chimeric *Smu*GalU-Δ294*Eco*GlgC enzyme were determined by gel filtration using a Tricorn 5/200 column (GE Healthcare). A Gel Filtration Calibration Kit-High Molecular Weight (GE Healthcare) with protein standards including thyroglobulin (669 kDa), ferritin (440 kDa), aldolase (158 kDa), conalbumin (75 kDa) and ovoalbumin (44 kDa) was used. The column void volume was determined using a dextran blue loading solution (Promega, Fitchburg, WI, USA).

### 3.9. Enzyme Assays

Synthesis of UDP-Glc was assayed by following the formation of P_i_ (after hydrolysis of PP_i_ by inorganic pyrophosphatase) by the highly sensitive colorimetric method previously described [41]. The standard reaction mixture contained 100 mM MOPS (pH 8.0), 3 mM MgCl_2_, 1.5 mM UTP, 0.2 mg/mL bovine serum albumin, 0.5 mU/μL yeast inorganic pyrophosphatase and a proper enzyme dilution. Assays were initiated by the addition of Glc-1P in a total volume of 50 μL. Reaction mixtures were incubated for 10 min at 37 °C and terminated by adding the Malachite Green reactive [41]. The complex formed with the released P_i_ was measured at 630 nm with an ELISA EMax detector (Molecular Devices) and using (sodium PP_i_) as standard.

One unit (U) of enzyme activity is equal to 1 μmol of product formed per minute under the respective assay conditions specified above.

### 3.10. Calculation of Kinetic Constants

Kinetic assays were performed using specified concentrations and conditions for all reaction mixture components. Saturation curves were performed by assaying the respective enzyme activity at saturating level of a fixed substrate and different concentrations of the variable substrate. The experimental data were plotted as enzyme activity (U/mg) *versus* substrate (or effector) concentration (mM), and kinetic constants were determined by fitting the data to the Hill equation as described elsewhere [20]. Fitting was performed with the Levenberg-Marquardt nonlinear least-squares algorithm provided by the computer program Origin™. Hill plots were used to calculate the Hill coefficient (*n*_H_), the maximal velocity (*V*_max_), and the kinetic constants that correspond to the activator, substrate or inhibitor concentrations giving 50% of the maximal activation (*A*_0.5_), velocity (*S*_0.5_) or inhibition (*I*_0.5_). All kinetic constants are the mean of at least three sets of data, which were reproducible within ±10%.

### 3.11. Homology Modeling

Modeling of the chimeric enzyme was performed using the program Modeller 9v1 [42]. For that purpose, the structure was modeled using four simultaneous templates; the known atomic coordinates of the UDP-Glc PPase from *C. glutamicum* in complex with magnesium and UDP-Glc (Chain A, PDB code 2pa4), the apo structure of the UDP-Glc PPase from *H. pylori* (chain A, PDB code 3juj), the structure of the ADP-Glc PPase from *A. tumefaciens* (PDB code 3brk), and the structure of potato tuber ADP-Glc PPase in complex sulfates in the putative regulatory site. In this way, the coordinates of the UDP-Glc PPases provide the structural information to build the *S. mutans* domain of the chimeric enzyme (N-terminus), and the ADP-Glc PPases the information needed to build the *E. coli* domain of the chimeric (C-terminus). The *A. tumefaciens* structure is particularly good for this, since it is a bacterial enzyme like *E. coli*, but the potato tuber structure provides information about where the sulfate ligands will go. Similarly, the *C. glutamicum* was used to place the product UDP-Glc in the model. The reliability of the model was evaluated using the programs Verify3D [43]. All the templates were manually structurally aligned to each other before the sequence alignment was performed with the chimeric target. The validation of the model with Verify3D was already good and further iterations of the alignment were not necessary.

## 4. Conclusions

Herein, we present results that strongly support that fusion of the C-terminal domain of *E. coli* ADP-Glc PPase to the entire UDP-Glc PPase from *S. mutans* produces a chimeric protein with UDP-Glc PPase activity sensitive to allosteric activation by specific metabolites. To the best of our knowledge this is the first report on allostery by binding of small molecules to a UDP-Glc PPase (even more in an NDP-Glc PPase distinct of ADP-Glc PPase) from prokaryotic or eukaryotic origin [5]. Previous work has demonstrated that UDP-Glc PPases are poorly or not regulated, with exceptions of: (i) The barley enzyme, the activity of which is affected by the occurrence of different quaternary arrangements, being active as a monomer [44–46]; and (ii) the *E. histolytica* enzyme that responds to regulation after modification by oxidants and reducing agents of critical cysteine residues [7].

ADP-Glc PPases are allosteric enzymes catalyzing the key step in glycogen and starch synthesis in bacteria and plants, respectively [1,2]. Many studies have been conducted in order to identify the amino acidic residues involved in the allosteric response. Thus, it has been proposed that Lys^39^ is important in the interaction with Fru-1,6-bisP with *E. coli* ADP-Glc PPase [47–49]. In addition, Arg^32^ from *A. tumefaciens* ADP-Glc PPase (similar to Lys^39^ in the *E. coli* enzyme), Arg^33^, and Arg^45^ are involved in allosteric effector binding [21]. In this case, it can be seen that these residues belong to the N-terminal domain of the bacterial ADP-Glc PPase, and a recent study has clearly established the importance of the *E. coli* ADP-Glc PPase *N*-terminal domain in the activation of the enzyme [22]. In addition, results where the first amino acidic residues from the N-terminal domains were also removed showed the enzyme to be fully active without the allosteric activator [50,51] However, results obtained with chimeric enzymes obtained after switching the N- and C-terminal domains from *E. coli* and *A. tumefaciens* ADP-Glc PPases indicated that the C-terminal region was critical in determining the affinity and specificity to effectors [19,22]. This also suggests that in prokaryotic ADP-Glc PPases both *N*- and *C*-terminal domains are interconnected in the response of the enzyme to the allosteric effectors. In this context, our results with the chimeric enzyme *Smu*GalU-Δ294*Eco*GlgC support this model, since we clearly demonstrated that the presence of a single C-terminal domain causes sensitivity to an allosteric activator to a previously non-allosteric UDP-Glc PPase.

It has to be remarked that the activation pattern exhibited by chimeric *Smu*GalU-Δ294*Eco*GlgC is a novel feature acquired by the *S. mutans* UDP-Glc PPase after being transformed (by domain fusion) in the hybrid enzyme. The latter strongly supports the functional allosteric regulatory role of the *C*-terminal domain found in ADP-Glc PPase. Results also support the view that such a domain can be modularly fused to add allosteric properties to different PPases. It is tempting to speculate that evolution followed a similar strategy to modify one enzymes of such type (with specificity toward ADP-Glc), elongating the protein to trigger allosteric regulation in a general way. Later, critical changes in the C regulatory domain would confer specificity for different allosteric effectors within ADP-Glc PPases. We do not know the mechanism by which the N-terminal domain is activated in presence of the C-terminal domain and the proper activator, but our experiments indicate that the catalytic domain of NDP-Glc PPases may have an intrinsic property to have their activity modulated in presence of certain interactions, even when they are not natively regulated. In this case, the presence of a foreign domain triggers the allosteric properties.

## Supplementary Information



## Figures and Tables

**Table 1:** Kinetic parameters for *Smu*GalU and *Smu*GalU-Δ294*Eco*GlgC. Parameters were calculated from averaged data from three independent experiments, as detailed in the Experimental Section.

Substrate	Kinetic parameter	*Smu*GalU	*Smu*GalU-Δ294*Eco*GlgC
	*V*_max_ (U/mg)	62.1 ± 2.2	15.4 ± 0.5

UTP	*S*_0.5_ (mM)	0.68 ± 0.06	0.24 ± 0.02
	*n*_H_	1.5 ± 0.2	1.2 ± 0.1
*V*_max_/*S*_0.5_ (U/mg mM)		91.3	64.2

Glc-1P	*S*_0.5_ (mM)	0.090 ± 0.005	0.060 ± 0.005
	*n*_H_	1.3 ± 0.1	1.4 ± 0.1
*V*_max_/*S*_0.5_ (U/mg mM)		690	257

Mg^2+^	*S*_0.5_ (mM)	0.81 ± 0.06	0.62 ± 0.05
	*n*_H_	2.4 ± 0.6	2.7 ± 0.6
*V*_max_/*S*_0.5_ (U/mg mM)		76.7	24.8

**Table 2:** Oligonucleotide primers employed to amplify *SmugalU* gene and to construct the gene coding for the *Smu*GalU-Δ294*Eco*GlgC chimeric protein. Restriction sites are underlined.

Primer	Sequence	Restriction site
**Smu1**	5′-GGATCCCATGCCAAGTAAAAAAGTCAG-3′	*Bam*HI
**Smu2**	5′-GAATTCCTTAATCCGAGTTCTTTTGAG-3′	*Eco*RI
**Qmr1**	5′-CTCGGACCCGGAACTGGATATGTACGATC-3′	--
**Qmr2**	5′-GTTCCGGGTCCGAGTTCTTTTGAGTCG-3′	--
**Qmr3**	5′-CTCGAGTTATCGCTCCTGTTTATGCCC-3′	*Xho*I
