# Supplementary material for: A Chimeric UDP-Glucose Pyrophosphorylase Produced by Protein Engineering Exhibits Sensitivity to Allosteric Regulators
Source: Int J Mol Sci. 2013 May 6;14(5):9703–21. doi: 10.3390/ijms14059703 (PMC3676807; doi:10.3390/ijms14059703)
Supplement: Supplementary file 1 [file ijms-14-09703-s001.pdf]

Supplementary Information

Figure S1. Alignment between different ADP-Glc PPases and UDP-Glc PPases Schematic, used in Panel A of Figure 1. (Ref: *Solanum*: ADP-Glc PPase small subunit from potato; *Atu*GlgC, *A. tumefaciens* ADP-Glc PPase; *Eco*GlgC, *E. coli* ADP-Glc PPase; Chimera, chimeric *Smu*GalU-Δ294*Eco*GlgC; *Smu*GalU, *S. mutans* UDP-Glc PPase; *Hpy*GalU, *H. pylory* UDP-Glc PPase; *Cgl*GalU, *C. glutamicum* UDP-Glc PPase). The amino acidic residue P^{295} indicating the beginning of the *E. coli* ADP-Glc PPase C-term domain is over-marked.

```
                  ....|....|....|....|....|....|....|....|....|....|....|....|
                           10        20        30        40        50        60
Solanum     (1)   --------QTCLDPDASRSVLGIILGGGAGTRLYPLTKKRAKPAVPLGANYRLIDIPVSN
AtuGlgC     (1)   --MSEK-----RVQPLARDAMAYVLAGGRGSRLKELTDRRAKPAVYFGGKARIIDFALSN
EcoGlgC     (1)   MVSLEKNDHLMLARQLPLKSVALILAGGRGTRLKDLTNKRAKPAVHFGGKFRIIDFALSN
CHIMERA     (1)   -------------MPSKKVRKAVIPAAGLGTRFLPATKALAKEMLPIV-DKPTIQFIVEE
SmuGalU     (1)   -------------MPSKKVRKAVIPAAGLGTRFLPATKALAKEMLPIV-DKPTIQFIVEE
HpyGalU     (1)   -----------------MIKKCLFPAAGYGTRFLPITKTIPKEMLPIV-DKPLIQYAVEE
CglGalU     (1)   -------MSLPIDEHVNAVKTVVVPAAGLGTRFLPATKTVPKELLPVV-DTPGIELIAAE

                  ....|....|....|....|....|....|....|....|....|....|....|....|
                           70        80        90       100       110       120
Solanum    (53)   CLNSNISKIYVLTQFNSASLNRHLSRAYASNMGGYKNEG---------------------
AtuGlgC    (54)   ALNSGIRRIGVATQYKAHSLIRHLQRGWDFFRPERNE-----------------------
EcoGlgC    (61)   CINSGIRRMGVITQYQSHTLVQHIQRGWSFFNEEMNE-----------------------
CHIMERA    (47)   ALKSGIEDILVVTGKSKRSIEDHFD--------------SNFELEYNLEQKGKTDLLKLV
SmuGalU    (47)   ALKSGIEDILVVTGKSKRSIEDHFD--------------SNFELEYNLEQKGKTDLLKLV
HpyGalU    (43)   AMEAGCEVMAIVTGRNKRSLEDYFD--------------TSYEIEHQIQGTNKENALKSI
CglGalU    (53)   AAELGATRLAIITAPNKAGVLAHFE--------------RSSELEETLMERGKTDQVEII

                  ....|....|....|....|....|....|....|....|....|....|....|....|
                          130       140       150       160       170       180
Solanum    (92)   -------FVEVLAAQQSPENPHWFQG-------TADAVRQYLWLFEEHTVLE--------
AtuGlgC    (91)   -------SFDILPASQRVSETQWYEG-------TADAVYQNIDIIEPYAPEY--------
EcoGlgC    (98)   -------FVDLLPAQQRMKGENWYRG-------TADAVTQNLDIIRRYKAEDILAGDH--
CHIMERA    (93)   NDTTAIN-LHFIRQS-----------HPRGLGDAVLQAK-------------AFVGNE--
SmuGalU    (93)   NDTTAIN-LHFIRQS-----------HPRGLGDAVLQAK-------------AFVGNE--
HpyGalU    (89)   RNIIEKCCFSYVRQK-----------QMKGLGHAILTGE-------------ALIGNE--
CglGalU    (99)   RRAADLIKAVPVTQD-----------KPLGLGHAVGLAE-------------SVLD--DD

                  ....|....|....|....|....|....|....|....|....|....|....|....|
                          190       200       210       220       230       240
Solanum   (130)   ---YLILAGDHLY-RMDYE----------KFIQAHRETDADITVAALPMDEKRATAFGLM
AtuGlgC   (129)   ---MVILAGDHIY-KMDYE----------YMLQQHVDSGADVTIGCLEVPRMEATGFGVM
EcoGlgC   (136)   ---VVILAGDHIY-KQDYS----------RMLIDHVEKGARCTVACMPVPIEEASAFGVM
CHIMERA   (126)   --PFVVMLGDDLMD-----ITDDKAIPLTRQLMNDYEETHA-STIAVMEVPHEDVSAYGV
SmuGalU   (126)   --PFVVMLGDDLMD-----ITDDKAIPLTRQLMNDYEETHA-STIAVMEVPHEDVSAYGV
HpyGalU   (123)   --PFAVILADDLC------ISHDHPSVLK-QMTSLYQKYQC-SIVAIEEVALEEVSKYGV
CglGalU   (133)   EDVVAVMLPDDLV------LPT---GVME-RMAQVRAEFGG-SVLCAVEVSEADVSKYGI

                  ....|....|....|....|....|....|....|....|....|....|....|....|
                          250       260       270       280       290       300
Solanum   (176)   KIDEE------------GRIIEFAEKPQGEQLQAMKVDTTILGLDDKRAKEM--------
AtuGlgC   (175)   HVNEK------------DEIIDFIEKP------------------------ADPPGIPGN
EcoGlgC   (182)   AVDEN------------DKIIEFVEKP------------------------ANPPSMPND
CHIMERA   (178)   IAP--QGEGVS----GLYSVDTFVEKP------------------------AP-------
SmuGalU   (178)   IAP--QGEGVS----GLYSVDTFVEKP------------------------AP-------
HpyGalU   (173)   IRG---EWLEE----GVYEIKDMVEKP------------------------NQ-------
CglGalU   (182)   FEI---EAD--TKDSDVKKVKGMVEKP------------------------AI-------

                  ....|....|....|....|....|....|....|....|....|....|....|....|
                          310       320       330       340       350       360
Solanum   (216)   --P-----FIASMGIYVISKDVMLNLLRDKF-PGANDFGSEV--------------IPGAT
```

Figure S1. *Cont.*

```
AtuGlgC   (199) EGF-----ALASMGIYVFHTK-FLMEAVRRDAA---DPTS--SRDFGK------DIIPYIV
EcoGlgC   (206) PSK-----SLASMGIYVFDAD-YLYELLEEDDR---DENS--SHDFGK------DLIPKIT
CHIMERA   (201) ---KEAPSNLAIIGRYLLT--PEIFTILET------------QEP-GAGNEVQLTDAIDTL
SmuGalU   (201) ---KEAPSNLAIIGRYLLT--PEIFTILET------------QEP-GAGNEVQLTDAIDTL
HpyGalU   (195) ---EDAPSNLAVIGRYILT--PDIFEILSE------------TKP-GKNNEIQITDALRTQ
CglGalU   (206) ---EDAPSRLAATGRYLLD--RKIFDALRR------------ITP-GAGGELQLTDAIDLL

                ....|....|....|....|....|....|....|....|....|....|....|....|
                        370       380       390       400       410       420
Solanum   (251) SLGMRVQAYLY------------DG--YWEDIGTIEAFYNANLGITK---KPV--------
AtuGlgC   (243) EHG-KAVAHRFADSCVRSDFEHE-P--YWRDVGTIDAYWQANIDLTD---V-V--------
EcoGlgC   (250) EAG-LAYAHPFPLSCVQSDPDAE-P--YWRDVGTLEAYWKANLDLAS---V-V--------
CHIMERA   (244) NKTQ-RVFAREF-------------KGKRYDVGDKFGFMKTSIDYALK-------------
SmuGalU   (244) NKTQ-RVFAREF-------------KGKRYDVGDKFGFMKTSIDYALK-------------
HpyGalU   (238) AKRK-RIIAYQF-------------KGKRYDCGSVEGYIEASNAYYKKRL-----------
CglGalU   (249) IDEGHPVHIVIH--------------QGKRHDLGNPGGYIPACVDFGLS-----HPVYGAQ

                ....|....|....|....|....|....|....|....|....|....|....|....|
                        430       440       450       460       470       480
Solanum   (291) -----------------------------------------PDFSFYDRSAPIYTQPRYLP
AtuGlgC   (288) -----------------------------------------PDLDIYDKSWPIWTYAEITP
EcoGlgC   (295) -----------------------------------------PELDMYDRNWPIRTYNESLP
CHIMERA   (280) --------------QVKEDLKAYIIELGKKLDQKSTQKNSDPELDMYDRNWPIRTYNESLP
SmuGalU   (280) --------------QVKEDLKAYIIELGKKLDQKSTQKNSD--------------------
HpyGalU   (274) -------------------------------------------------------------
CglGalU   (291) LKDAIKQILAEHEAA----------------------------------------------

                ....|....|....|....|....|....|....|....|....|....|....|....|
                        490       500       510       520       530       540
Solanum   (311) SSKML--------DADVTDSVIGEGCVIKNCKIHHSVVGLRSCISEGAIIEDSLLMG—ADY
AtuGlgC   (308) PAKFVHDDEDRRG—SAVSSVVSGDCIISGAALNRSLLFTGVRANSYSRLENAVVLPSV---
EcoGlgC   (315) PAKFVQDRSGSHG—MTLNSLVSGGCVISGSVVVQSVLFSRVRVNSFCNIDSAVLLPEV---
CHIMERA   (327) PAKFVQDRSGSHG—MTLNSLVSGGCVISGSVVVQSVLFSRVRVNSFCNIDSAVLLPEV---
SmuGalU   (307) -------------------------------------------------------------
HpyGalU   (274) -------------------------------------------------------------
CglGalU   (305) -------------------------------------------------------------

                ....|....|....|....|....|....|....|....|....|....|....|....|
                        550       560       570       580       590       600
Solanum   (363) YETDADRKLLAAKGSVPIGIGKNCHIKRAIIDKNARIGDNVKII----------EAARETD
AtuGlgC   (365) ------------------KIGRHAQLSNVVIDHGVVIPEGLIVGEDPELDAKRFRRTESGI
EcoGlgC   (372) ------------------WVGRSCRLRRCVIDRACVIPEGMVIGENAEEDARRFYRSEEGI
CHIMERA   (383) ------------------WVGRSCRLRRCVIDRACVIPEGMVIGENAEEDARRFYRSEEGI
SmuGalU   (307) -------------------------------------------------------------
HpyGalU   (274) -------------------------------------------------------------
CglGalU   (305) -------------------------------------------------------------

                ....|....| ....|....|
                        610       620
Solanum   (420) GYFIKSGIVTVIKDALIPSGIVI
AtuGlgC   (365) CLITQSMIDKLDL----------
EcoGlgC   (415) VLVTREMLRKLGHKQER------
CHIMERA   (427) VLVTREMLRKLGHKQER------
SmuGalU   (307) -----------------------
HpyGalU   (274) -----------------------
CglGalU   (305) -----------------------
```

Figure S2. Alignment of chimeric enzyme sequence and templates used for homology modeling. (Ref.: 2pa4a, chain A of *C. glutamicum* UDP-Glc PPase; 3juja, chain A of *H. pylori* UDP-Glc PPase; 3BRK, *A. tumefaciens* ADP-Glc PPase; 1yp3a, chain A of potato tuber ADP-Glc PPase).

```
                ....|....| ....|....| ....|....| ....|....| ....|....| ....|....|
                        10         20         30         40         50         60
2pa4a           ---------- VKTVVVPAAG LGTRFLPATK TVPKELLPVV -DTPGIELIA AEAAELGATR
3juja           ---------M IKKCLFPAAG YGTRFLPITK TIPKEMLPIV -DKPLIQYAV EEAMEAGCEV
3BRK            -----QPLAR DAMAYVLAGG RGSRLKELTD RRAKPAVYFG GKARIIDFAL SNALNSGIRR
1yp3a           QTCLDPDASR SVLGIILGGG AGTRLYPLTK KRAKPAVPLG ANYRLIDIPV SNCLNSNISK
Chimeric        -----MPSKK VRKAVIPAAG LGTRFLPATK ALAKEMLPIV -DKPTIQFIV EEALKSGIED

                ....|....| ....|....| ....|....| ....|....| ....|....| ....|....|
                        70         80         90        100        110        120
2pa4a           LAIITAPNKA GVLAHFE--- ---------R SSELEETLME RGKTDQVEII RRAADLIKAV
3juja           MAIVTGRNKR SLEDYFD--- ---------T SYEIEHQIQG TNKENALKSI RNIIEKCCFS
3BRK            IGVATQYKAH SLIRHLQRGW DFFRPERNE- ---------- ---------- -------SFD
1yp3a           IYVLTQFNSA SLNRHLSRAY -------EG- ---------- ---------- -------FVE
Chimeric        ILVVTGKSKR SIEDHFD--- ---------S NFELEYNLEQ KGKTDLLKLV NDTTAIN-LH

                ....|....| ....|....| ....|....| ....|....| ....|....| ....|....|
                       130        140        150        160        170        180
2pa4a           PVTQD----- ------KPLG LGHAVGLAE- ---------- --SVLD--DD EDVVAVMLPD
3juja           YVRQK----- ------QMKG LGHAILTGE- ---------- --ALIGNE-- --PFAVILAD
3BRK            ILPAS----- --WYEG---- ---TADAVYQ NIDIIEPYAP EY-------- ---MVILAGD
1yp3a           VLAAQQSPEN PDWFQG---- ---TADAVRQ YLWLFEEHTV LE-------- ---YLILAGD
Chimeric        FIRQS----- ------HPRG LGDAVLQAK- ---------- --AFVGNE-- --PFVVMLGD

                ....|....| ....|....| ....|....| ....|....| ....|....| ....|....|
                       190        200        210        220        230        240
2pa4a           DLV------L PT---GVME- RMAQVRAEFG G-SVLCAVEV SEADVSKYGI FEI----EAD
3juja           DLC------I SHDHPSVLK- QMTSLYQKYQ C-SIVAIEEV ALEEVSKYGV IRG----EWL
3BRK            HIY-KMDYE- ---------- YMLQQHVDSG ADVTIGCLEV PRMEATGFGV MHVNEK----
1yp3a           HLY-RMDYE- ---------- KFIQAHRETD ADITVAALPM DEKRATAFGL MKIDEE----
Chimeric        DLMD-----I TDDKAIPLTR QLMNDYEETH A-STIAVMEV PHEDVSAYGV IAP---QGEG

                ....|....| ....|....| ....|....| ....|....| ....|....| ....|....|
                       250        260        270        280        290        300
2pa4a           --TKDSDVKK VKGMVEKP-- ---------- ---------- ---AI----- -----EDAPS
3juja           EE----GVYE IKDMVEKP-- ---------- ---------- ---NQ----- -----EDAPS
3BRK            --------DE IIDFIEKP-- ---------- ---------- ---ADPPGIP GNEGF-----
1yp3a           --------GR IIEFAEKPQG EQLQAMKVDT TILGLDDKRA KEM------- ----P-----
Chimeric        VS----GLYS VDTFVEKP-- ---------- ---------- ---AP----- -----KEAPS

                ....|....| ....|....| ....|....| ....|....| ....|....| ....|....|
                       310        320        330        340        350        360
2pa4a           RLAATGRYLL D--RKIFDAL RR-------- -----ITPGA GGELQLTDAI DLLIDEGHPV
3juja           NLAVIGRYIL T--PDIFEIL SE-------- -----TKPGK NNEIQITDAL RTQAKRK-RI
3BRK            ALASMGIYVF HTK-FLMEAV RRDAA----- ---DI----- ---------I PYIVEHG-KA
1yp3a           FIASMGIYVI SKD-VMLNLL RDKF-PGAND FGSEV----- ---------I PGATSLGMRV
Chimeric        NLAIIGRYLL T--PEIFTIL ET-------- -----QEPGA GNEVQLTDAI DTLNKTQ-RV

                ....|....| ....|....| ....|....| ....|....| ....|....| ....|....|
                       370        380        390        400        410        420
2pa4a           HIVIH----- ---------Q GKRHDLGNPG GYIPACVDFG LS-----HPV YGAQLKDAIK
3juja           IAYQF----- ---------K GKRYDCGSVE GYIEASNAYY KKRL------ ----------
3BRK            VAHRFADSCV RSDFEHE-P- -YWRDVGTID AYWQANIDLT D---V-V--- ----------
1yp3a           QAYLY----- -------DG- -YWEDIGTIE AFYNANLGIT K---KPV--- ----------
Chimeric        FAREF----- ---------K GKRYDVGDKF GFMKTSIDYA LKHP------ ----------

                ....|....| ....|....| ....|....| ....|....| ....|....| ....|....|
```

Figure S2. *Cont.*

```
                  430        440        450        460        470        480
2pa4a      QILAEHEAA- ---------- ---------- ---------- ---------- ----------
3juja      ---------- ---------- ---------- ---------- ---------- ----------
3BRK       ---------- ---------- ---------- ------PDLD IYDKSWPIWT YAEITPPAKF
1yp3a      ---------- ---------- ---------- ------PDFS FYDRSAPIYT QPRYLPPSKM
Chimeric   ---------Q VKEDLKAYII ELGKKLDQKS TQKNSDPELD MYDRNWPIRT YNESLPPAKF

           ....|....| ....|....| ....|....| ....|....| ....|....| ....|....|
                  490        500        510        520        530        540
2pa4a      ---------- ---------- ---------- ---------- ---------- ----------
3juja      ---------- ---------- ---------- ---------- ---------- ----------
3BRK       VHDDEDRRG- -SAVSSVVSG DCIISGAALN RSLLFTGVRA NSYSRLENAV VLPSV-----
1yp3a      L--------D ADVTDSVIGE GCVIKNCKIH HSVVGLRSCI SEGAIIEDSL LMG--ADYYE
Chimeric   VQDRSGSHG- -MTLNSLVSG GCVISGSVVV QSVLFSRVRV NSFCNIDSAV LLPEV-----

           ....|....| ....|....| ....|....| ....|....| ....|....| ....|....|
                  550        560        570        580        590        600
2pa4a      ---------- ---------- ---------- ---------- ---------- ----------
3juja      ---------- ---------- ---------- ---------- ---------- ----------
3BRK       ---------- ------KIGR HAQLSNVVID HGVVIPEGLI VGEDPELDAK RFRRTESGIC
1yp3a      TDADRKLLAA KGSVPIGIGK NCHIKRAIID KNARIGDNVK II-------- ----------
Chimeric   ---------- ------WVGR SCRLRRCVID RACVIPEGMV IGENAEEDAR RFYRSEEGIV

           ....|....| ....|....| ....|....| ....|....| ....|....| ..
                  610        620        630        640        650
2pa4a      ---------- ---------- ---------- ---------- ---------- --
3juja      ---------- ---------- ---------- ---------- ---------- --
3BRK       LITQSMIDKL ---------- ---------- ---------- ---------- --
1yp3a      ---------- ------NKDN VQEAARETDG YFIKSGIVTV IKDALIPSGI II
Chimeric   LVTREMLRKL GHKQER---- ---------- ---------- ---------- --
```

© 2013 by the authors; licensee MDPI, Basel, Switzerland. This article is an open access article distributed under the terms and conditions of the Creative Commons Attribution license (http://creativecommons.org/licenses/by/3.0/).
